# Impact of a decision aid on reducing uncertainty: pilot study of women in their 40s and screening mammography

**DOI:** 10.1186/s12911-015-0210-2

**Published:** 2015-11-10

**Authors:** Paula Scariati, Lisa Nelson, Lindsey Watson, Stephen Bedrick, Karen B. Eden

**Affiliations:** Marin General Hospital, Greenbrae, CA USA; ConvergeHEALTH by Deloitte, Newton, MA USA; Department of Medical Informatics and Clinical Epidemiology, Oregon Health & Science University, Portland, OR USA; Center for Spoken Language and Understanding, Oregon Health & Science University, Portland, OR USA; Pacific Northwest Evidence-based Practice Center, Oregon Health & Science University, Portland, OR USA

**Keywords:** Decision support techniques, Mammography, Screening, Cancer prevention, Patient preferences, Decision aid

## Abstract

**Background:**

In 2009 the United States Preventive Services Task Force updated its breast cancer screening guidelines to recommend that average-risk women obtain a screening mammogram every two years starting at age 50 instead of annually starting at age 40. Inconsistencies in data regarding the benefit versus risk of routine screening for women less than 50-years-of-age led to a second recommendation – that women in their forties engage in a shared decision making process with their provider to make an individualized choice about screening mammography that was right for them. In response, a web-based interactive mammography screening decision aid was developed and evaluated.

**Methods:**

The decision aid was developed using an agile, iterative process. It was further honed based on feedback from clinical and technical subject matter experts. A convenience sample of 51 age- and risk-appropriate women was recruited to pilot the aid. Pre-post decisional conflict and screening choice was assessed.

**Results:**

Women reported a significant reduction in overall decisional conflict after using the decision aid (Z = -5.3, *p* < 0.001). These participants also reported statistically significant reductions in each of the decisional conflict subscales: feeling uncertain (Z = -4.7, *p* < 0.001), feeling uninformed (Z = -5.2, *p* < 0.001), feeling unclear about values (Z = -5.0, *p* < 0.001), and feeling unsupported (Z = -4.0, *p* < 0.001). However, a woman’s intention to obtain a screening mammogram in the next 1-2 years was not significantly changed (Wilcoxon signed-rank Z = -1.508, *p* = 0.132).

**Conclusion:**

This mammography screening decision aid brings value to patient care not by impacting what a woman chooses but by lending clarity to *why* or *how* she chooses it.

**Electronic supplementary material:**

The online version of this article (doi:10.1186/s12911-015-0210-2) contains supplementary material, which is available to authorized users.

## Background

Concern and disagreement was voiced when the United States Preventive Services Task Force (USPSTF) updated its breast cancer screening guidelines in November 2009 [1–6]. The guidelines transitioned from recommending that average-risk women obtain a screening mammogram yearly starting at age 40 to now undergoing screening every two years starting at age 50 [7, 8]. In the absence of data to support the benefit versus harm of routine screening, average-risk women in their 40s were advised to engage in an individual shared decision making process with their provider to determine if screening was right for them [7, 8].

Shared decision making is a process where the patient and clinician share information with each other, assess evidence and values, and mutually agree upon a course of action. The goal is to create the *activated patient* - a patient who is well-informed about the benefits and harms of a particular issue and feels comfortable that her choice reflects the appropriate combination of clinical input, evidence and personal values [9, 10]. Shared decision making is ideal when the tradeoff between benefits and harms is either unclear or unknown.

A recently updated Cochrane review on decision making concluded that decision aids are better than usual care interventions in providing patients with knowledge and reducing conflict about making a decision [11]. Patients have a more realistic expectation of benefits and harms as well as the values surrounding them. They also activate undecided or passive patients to make a decision. For example, exposure to these tools resulted in patients choosing more conservative surgery options and reduced prostate specific antigen (PSA) screening [11].

There are a wide variety of decision making resources that can be used to facilitate a shared decision making process. At one end of the spectrum, a decision aid can be a simple, patient-focused brochure that explains the benefits and harms of a specific procedure. On the other end of the spectrum, it can be a web-based, interactive tool that engages the patient to respond to specific questions while providing targeted information and feedback based on probabilistic models [12–14]. In this project the goal was development and evaluation of a web-based decision aid that helped average-risk women in their 40s understand, rank and sort their values regarding screening mammography. At the end of this process the woman was presented with a summary document intended to facilitate a shared decision making conversation between her and her provider.

This manuscript explores the decision aid development process, refinement through iterative feedback, and performance in a pilot cohort. The aims of this pilot study were 1) to explore the impact of a web-based patient decision aid on a woman’s decisional conflict about the screening mammography decision, and 2) to explore their values related to screening mammography through an explicit values-clarification exercise included in a patient decision aid.

### Significance

Breast cancer is the most common cancer diagnosed in women and the second leading cause of cancer death [15]. There is no effective way to prevent breast cancer which is why the appropriate application of screening mammography is critical. The benefits of having a screening mammogram must outweigh the harms of not having the test or of having a test result that is falsely positive. This is known as net benefit. Conflicting data about whether average-risk women in their 40s experience a net benefit from routine screening mammograms led the USPSTF to recommend that they engage in an individualized shared decision making process. The goal was to empower these women to make a screening decision that is right for them.

## Methods

### Decision aid description

**Fig. 1:**
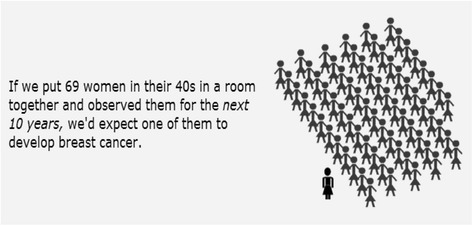
Current breast cancer risk. Infographic depicting current breast cancer risk statistic for average-risk women in their 40s

The decision aid was organized into six sections and the content was guided by International Patient Decision Aid Standards (IPDAS) collaboration criteria [16]: (1) welcome, (2) breast cancer risk factors, (3) mammography information, (4) values clarification, (5) summary and (6) final questions. The welcome section defined screening, contrasted diagnostic and screening mammography, and explored the population risk of breast cancer using pictographs (Fig. 1). The risk factors section explored most modifiable and non-modifiable risk factors for breast cancer development. The mammography section described the screening process, safety of it, timing between mammography screenings, and the possible incorrect outcomes of a screening mammogram.

The values clarification section was comprised of two exercises. First, the woman was asked to slide an indicator box on a series of scale bars (see Additional file 1) to reflect the importance of ten factors on her decision to having a screening mammogram: avoiding false positives, avoiding false negatives, peace of mind, catching cancer early, overdiagnosis, stress & fear, time & access, embarrassment & pain, radiation exposure and cost. The factors chosen for assessment reflected major concerns raised in the biomedical literature and vetted with subject matter experts, described in the *Decision Aid Development* section. The women could not add additional factors.

The goal was for the woman to interpret the information presented in light of her personal values and circumstances. For example, one questions asked was *“Which is more important to you, having a positive test when cancer is present or avoiding a positive test when cancer is not present?”* The far left hand side of the scale bar was labeled “*Having a Positive Test When Cancer is Present”* while the far right hand side of the scale was labeled “*Avoiding a Positive Test When There is No Cancer”.* The woman then moved the indicator box on the scale bar to the point that reflected her balance point in considering the risks versus benefits of these two possible outcomes.

**Fig. 2:**
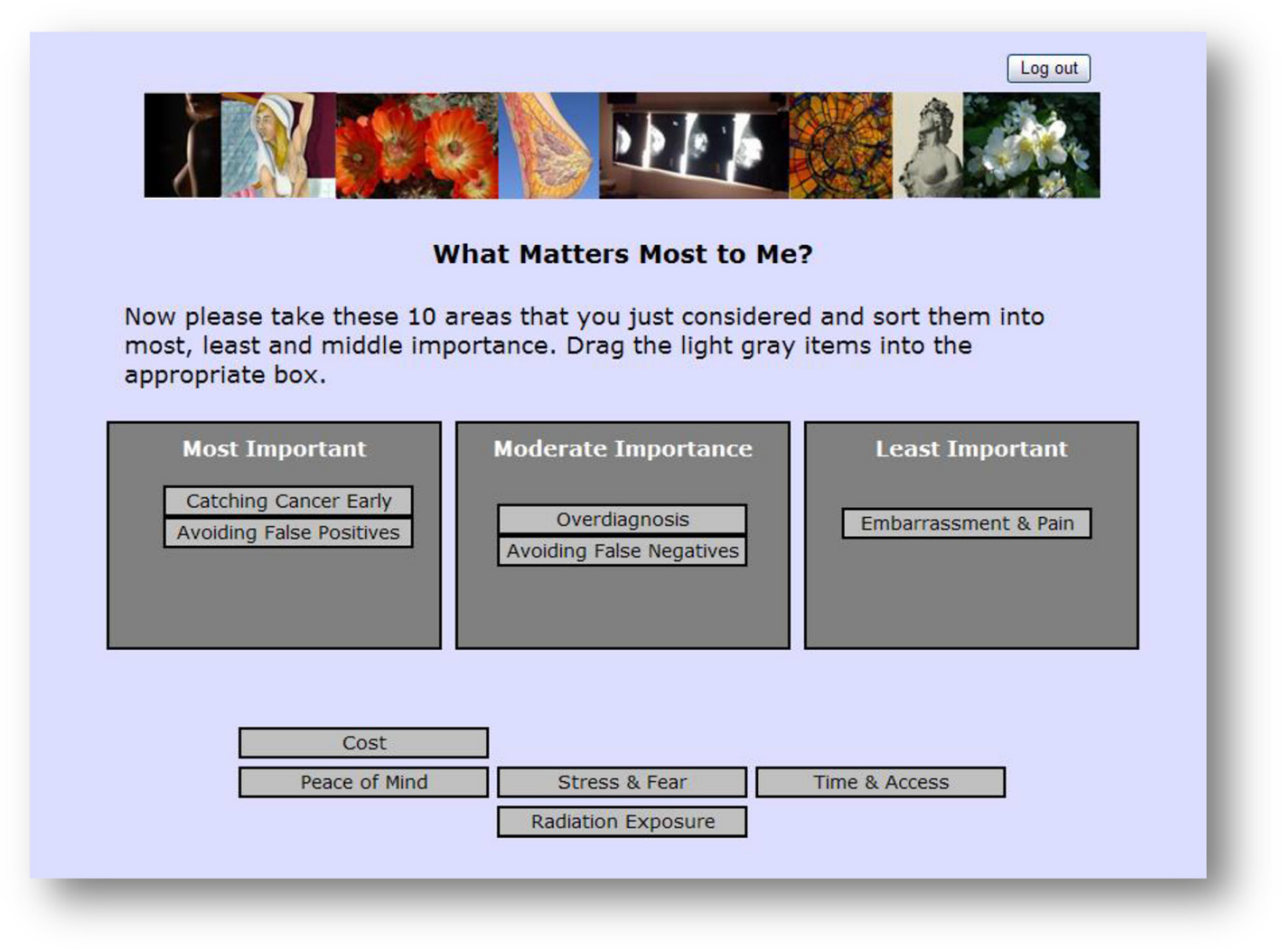
Values clarification. Ranking factors to consider when making a decision about screening mammography, by importance

The second part of the values clarification exercise entailed ranking these ten factors into one of three categories: (1) most important, (2) moderate importance, or (3) least important (Fig. 2). In the process of performing this ranking exercise, a woman came to understand her expectations and concerns surrounding screening mammography. This information was then captured and included as part of a summary that the woman received when she completed the aid. The woman was encouraged to review this information and discuss it with her primary care provider as part of her individualized shared decision making process.

### Decision aid development

Several resources were leveraged for developing and refining the screening mammography decision aid throughout 2010 and into the first half of 2011. An extensive review of the biomedical literature was step one. This was used to determine the initial scope and content of the aid [7, 8, 17–19]. Several rounds of internal testing were conducted with informatics graduate students to assess the technical aspects and usability of the decision aid. Healthcare professional and graduate students also provided feedback on content, comprehensibility, length and adherence to privacy and security standards.

Before testing with patients, five subject matter experts from the USPSTF, National Cancer Institute (NCI), the Agency for Healthcare Research (AHRQ), the American Cancer Society (ACS) and the American College of Obstetricians and Gynecologists (ACOG) and five clinical experts in women’s health were interviewed for face validity and on their impression of and experience using the decision aid. The semi-structured interview questions probed issues such as whether the message for an ineligible (above-average-risk) woman was sensitive and encouraged her to follow-up with her own healthcare provider. The experts also identified ways to make the content and output useful for discussions with the woman’s healthcare provider.

This solicited feedback from subject matter and clinical experts during development was an unusual but valuable step in shaping this decision aid. It was key in honing tool content as well as raising and exploring unforeseen issues. Furthermore, it allowed for a better understanding of how to handle the epidemiologic divide that prevents the experts from agreeing on a single breast cancer screening recommendation.

Recommendations included replacing terms like “false negatives” with plain language such as, “results that say you don’t have cancer when you actually do.” Many experts also recommended enhancing decision tree diagrams with flash animations or explanatory text, and providing the user with hyperlinks to definitions of more complex words or potentially confusing concepts. Revisions were made to the decision aid to incorporate these suggestions.

The decision aid was password protected and accessible through web browsers including Mozilla Firefox and Microsoft Internet Explorer.

### Ethics and consent

This study was approved by the OHSU Research Integrity Office (eIRB #7118). All enrolled women completed informed consent. For confidentiality reasons, only the group results are presented and no additional supplementary data are available to researchers.

### Protocol and participants

In August 2011 social media (i.e., Facebook) and interpersonal networking (i.e., friend-to-friend) was leveraged to recruit women for this pilot project. Sixty-four women indicated their desire to participate in the pilot by sending an email to the research team. In response, each woman was provided with a username, password and detailed instructions for accessing the aid. Subject eligibility was determined through a computerized risk assessment algorithm (described below). Six women were ineligible due to age or were above-average breast cancer risk; five enrolled but did not log into the tool; and two logged into the tool but did not complete the entire process. The remaining 51 age-risk appropriate women test piloted the decision aid. They received a $15.00 Starbuck’s Card eGift by email as a token of appreciation.

### Risk assessment

Women accessed the risk assessment on a personal computer through the Internet with a username and password. Once in the website, each woman engaged in a series of risk stratification questions aimed at identifying and restricting women with an increased risk of developing breast from engaging with the decision aid [20]. This algorithm included personal and family history of breast or ovarian cancer, genetic markers for breast cancer, current breast symptoms, or a prior history of radiation. Women with an above-average risk of developing breast cancer were directed to appropriate screening and follow-up in accordance with best practices. Average-risk women then moved on to use the decision aid.

### Measures and analyses

Women were asked baseline questions regarding their intention to obtain a screening mammogram in the next 1-2 years. They then completed a series of ten questions designed to assess their baseline decisional conflict around making a screening mammography choice (Table 1). This Decisional Conflict Scale (DCS) measured the amount of certainty, clarity of values, level of support and how informed a woman felt when making a decision about screening mammography. The DCS used here has been well-validated in the medical literature [21–23]. Next, each woman answered questions on basic demographic information including race, education level, income, and health insurance. In addition, she provided information on history of mammograms, prior false-positive screens and intention to have (or continue to have) screening mammograms.

**Table 1:** Modified low-literacy decision conflict scale

Participants answered *Yes, Unsure,* or *No* to each of the following questions:
1. Do you know what mammography screening options are available to you?
2. Do you know the benefits of each option?
3. Are you clear about which benefits matter most to you?
4. Do you know the risks and side effects of each option?
5. Are you clear about which risks and side effects matter most to you?
6. Do you have enough support from others to make a choice?
7. Are you choosing without pressure from others?
8. Do you have enough advice to make a choice?
9. Are you clear about the best choice for you?
10. Do you feel sure about what to choose?

The Decisional Conflict Scale [24], low literacy format, was adapted to measure the overall amount of conflict experienced when considering a decision about screening mammography. Scale subscores provided additional information on knowledge, values clarity, support and certainty

To further measure the specific impact of the decision aid on each woman’s decision making process, scores from the four subscales and summary scale of the low-literacy version of the DCS were calculated [24]. For each of ten statements, e.g., “Do you know what your options are for breast cancer screening?”, the patient responds with “Yes” (zero points), “No” (four points), or “Unsure” (two points). The mean score of the questions was determined for each subscale (feeling uninformed; feeling uncertain, having unclear values; feeling unsupported) and multiplied by 25. The mean score of all questions was also determined and multiplied by 25 to create a summary score. Summary scores of zero suggest an overall good decision process (no decisional conflict); scores of 100 suggest a poor decision process (extremely high decisional conflict). Improvement was measured by reduction in scores. This instrument was completed by all participants prior to engaging with the content of the decision aid and after completing their interaction with it. This facilitated discrimination between patients who make decisions and patients who delay making decisions because of uncertainty [24, 25].

### Sample size justification

Decisional conflict was the primary outcome assessed in this pilot study, and as such we looked to prior experimental studies to guide our plans regarding sample size. A similar pre-post study design, which evaluated a breast cancer prevention decision aid combined with counseling, enrolled 17 higher-risk women [25]. That study reported a reduction in overall decisional conflict, but the reductions were not statistically significant (*p* > 0.05) in any of the subscales, possibly due to the study’s limited sample size. The decision aid in our present study was designed for *average*-risk women, and it was not clear to us whether our study’s participants would have similar baseline levels of decisional conflict to those observed by Stacey [26] among higher-risk women. As such, we took a conservative approach and planned to recruit 50 women to participate in this pilot study.

Statistical analyses were carried out using IBM SPSS Statistics 19 (IBM Corporation, Somers, NY, USA) between September 30 and November 1, 2011. Descriptive statistics were calculated for all variables of interest. Continuous measures were summarized using means and standard deviations. Categorical measures were summarized using counts and percentages. The two main outcomes of interest, intention to obtain a screening mammogram and decisional conflict, were measured in a pre-post fashion. Because these variables were not normally distributed, a Wilcoxon signed-rank test was used to assess significance. A significance level of 0.05 was used unless multiple comparisons were indicated. In the latter case a significance level of 0.01 or appropriate correction (i.e., Bonferroni) was used.

## Results

A convenience sample of 51 women aged 38 - 48 with no known risk factors for developing breast cancer participated in this pilot study between August 22 and September 24, 2011. This cohort was predominantly white (84 %), well-educated (78 % with at least a college degree), insured (98 % had health insurance) and financially comfortable (45 % with an annual household income of at least $100,000) (Table 2). Thirty-eight women (74 %) reported having at least one prior mammogram with 19 of them (50 %) experiencing a false positive test result at some point in time (Table 2).

**Table 2:** Demographic characteristics of pilot study participants (*n* = 51)

Characteristic	n (%)
White	42 (83)
Asian	6 (12)
Black	2 (4)
More than 1	1 (2)
Education Level	
HS Diploma	1 (2)
Some College	10 (20)
College Degree	21 (41)
Some Graduate	7 (14)
Graduate Degree	12 (23)
Income	
10 K - <25 K	1 (2)
25 K - < 50 K	6 (12)
50 K - < 100 K	19 (37)
100 K or more	23 (45)
No Response	2 (4)
Health Insurance	
Yes	50 (98)
No	1 (2)
Prior Mammogram	
Yes	38 (74)
No	13 (26)
Prior False Positive Screen	
Yes	19 (37)
No	19 (37)
Not Applicable	13 (26)

This cohort was predominantly white (84 %), well-educated (78 % with at least a college degree), insured (98 % had health insurance) and financially comfortable (45 % with an annual household income of at least $100,000). Thirty-eight women (74 %) reported having at least one prior mammogram with 19 of them (50 %) experiencing a false positive test result at some point in time

When asked whether they planned to have a mammogram in the next 1-2 years, 42 women (82 %) said yes, 4 (8 %) were undecided and 5 (10 %) said no. At baseline, 13 women (25 %) experienced no decisional conflict (scored 0 for overall decisional conflict) and 6 (12 %) had some decisional conflict with scores between 1 and 25. Scores below 25 are associated with making decisions (O’Connor, decisional conflict manual, 2010). Ten women (21 %) had overall decisional conflict scores between 26 and 50; 14 (27 %) had had scores between 51 and 75; and 8 (16 %) had scores above 75. This suggests that the majority of women had scores over the threshold for making decisions and were likely to delay the decision [24].

Also at baseline, 45 women (88 %) indicated a belief that women in their 40s benefit from screening mammography and that the choice to have a screening mammogram should be something that they themselves decide (63 %) or a decision that is made together with their healthcare provider (37 %). No member of this cohort felt that a healthcare provider should make this decision for them or expressed uncertainty about who should make this choice.

A Wilcoxon signed-rank test was used to compare a woman’s plans for screening mammography before and after using the decision aid. After using the decision aid, women reported a significant reduction in overall decisional conflict (Z = -5.3, *p* < 0.001) as well as statistically significant reductions in each of the decisional conflict subscales: feeling uncertain (Z = -4.7, *p* < 0.001), feeling uninformed (Z = -5.2, *p* < 0.001), feeling unclear about values (Z = -5.0, *p* < 0.001), and feeling unsupported (Z = -4.0, *p* < 0.001) (Table 3). However, these women reported no change in screening intention (Z = -1.5, *p* = 0.132).

**Table 3:** Decisional conflict scale pre- and post-scores

Category	Pre-score	Std. Dev	Post-score	Std. Dev	Delta	Statistic^a^
Overall	40.6	31.0	6.4	13.1	−34.2	Z = -5.3, *p* < 0.001
Subscores						
Uncertain	50.5	41.4	12.8	27.6	−37.7	Z = -4.7, *p* < 0.001
Uninformed	47.4	38.2	3.9	14.8	−43.5	Z = -5.2, *p* < 0.001
Unclear values	51.0	42.7	5.9	21.0	−45.1	Z = -5.0, *p* < 0.001
Unsupported	20.3	22.2	4.9	10.7	−15.4	Z = -4.0, *p* < 0.001

^a^Wilcoxan Signed-Rank Test

Significant reduction was seen in overall decisional conflict scores, and in each of the decisional conflict subscores

**Fig. 3:**
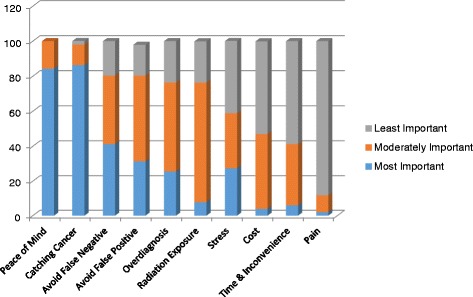
Patient personal values ranking results. Participants’ ratings of personal values when considering a decision about screening mammography, by importance

During the ranking exercise, women were asked to classify ten factors - time & access, peace of mind, embarrassment & pain, false positive test results, false negative test results, radiation exposure, cancer detection, overdiagnosis, stress & fear, and cost - into one of three buckets: most important, moderately important, or least important. Catching cancer earlier through screening was ranked most important by 44 (86 %) women. This was followed closely by peace of mind (84 %). Other factors ranked with high and moderate importance included avoiding false negative tests, avoiding false positive tests, overdiagnosis and radiation exposure (Fig. 3).

## Discussion

This pilot study of a breast cancer screening decision aid with a convenience sample of 51 age- and risk-appropriate women offers two main contributions. First, it demonstrates that decisional conflict about screening mammography may be significantly impacted by the use of a decision aid. Women in this pilot study felt more certain, better informed and better supported, and they demonstrated increased clarity of values in their decision making process. This finding is tempered by lack of a control condition and the fact that these women had no change in the intention to obtain a routine screening mammogram. This latter finding, while interesting, is not all together unexpected. Most (88 %) of the women in this sample held the baseline belief that women in their 40s benefit from screening mammography; 74 % had a prior mammogram; and 82 % planned to have a mammogram within the next year. This seems to reflect long-standing cultural and peer norms which one wouldn’t expect to shift readily or easily.

This absence of a change in intention to obtain a routine screening mammogram also raises the question of the clinical impact and value of this decision aid. Here we must be clear that the shared decision making process itself, initiated through this decision aid and independent of the final choice(s) made, has significant clinical value that should not be marginalized. This includes the benefits of patient engagement and satisfaction; standardization of best practice messaging; more realistic expectations of risks and harms; as well as a more efficient use of the clinician’s time.

The second contribution is the use of a values clarification technique involving rating the importance of selected factors that might impact the screening decision and then ranking those factors. More than 80 % of the women in this study ranked peace of mind and detecting a cancer early as the “most important” factors to consider when making a decision about screening mammography. This was followed by “most important” concerns around avoiding false negatives (41 %), avoiding false positive (31 %) and overdiagnosis (26 %). This seemed indicative of an understanding of these more nuanced terms as well as the risk-benefit associated with screening mammography. Less than 5 % of the women ranked cost as a “most important” factor. This may reflect the high income level of the group or the expanding knowledge that free screening mammography is readily available.

This pilot study contains limitations that can be lifted with future research. First, the decision aid was tested with a convenience sample of women who were predominantly white, well-educated and highly resourced. These women were not representative of the general population, and the findings from this study cannot be generalized. Second, the aid was available only in format requiring internet access. Users of iPads and smart phones were also excluded, though it should be noted that use of such devices was less common at the time the study was conducted than today. Finally, the aid may have been too long. During usability testing it took approximately 35 min to get through the entire decision aid.

The limitations identified in this pilot are now being addressed through a follow-on study funded by the McKesson Foundation’s *Mobilizing For Health Grant Program*. The decision aid has been redesigned as an app on a mobile device. The target audience for the app is low-income, average-risk women in their forties, who are not regularly screening and who seek care in rural clinics. As with this pilot, the women enrolled in the follow-on study also undergo a breast cancer risk assessment to confirm eligibility. Above-average-risk women are then given resources to help them obtain timely and appropriate follow-up screening, and average-risk women are invited to engage with the decision aid and make an informed choice based on their own preferences or priorities.

## Conclusion

This breast cancer screening decision aid brought value to patient care not by impacting *what* a woman chose, but by impacting *why* or *how* she chose it. There are several ways to decrease conflict, including becoming informed about choices for screening; feeling supported in the screening decision; knowing personal priorities around the decision; and feeling certain about the decision. The decision aid described in this pilot study addressed all of these aspects of the decision making process.

## Additional file

Additional file 1:
**Screen shots depicting the Values Clarification section of the Mammography Screening Decision Aid.** (PDF 332 kb)

## References

[1] Murphy AM (2010). Mammography screening for breast cancer: a view from 2 worlds (Editorial). JAMA.

[2] Woolf SH (2010). The 2009 breast cancer screening recommendation of the US Preventive Services Task Force (Editorial). JAMA.

[3] DeAngelis CD, Fontanarosa PB (2010). U.S. Preventive Services Task Force and breast cancer screening (Editorial). JAMA.

[4] Keen JD (2010). Promoting screening mammography: insight or uptake?. J Am Board Fam Med.

[5] Hirsch BR, Lyman GH (2011). Breast cancer screening with mammography. Curr Oncol Rep.

[6] American Cancer Society (2009). American Cancer Society responds to changes to USPSTF mammography guidelines.

[7] U.S. Preventive Services Task Force (USPSTF) (2009). Screening for breast cancer: U.S. Preventive Services Task Force recommendation statement. Ann Intern Med.

[8] Nelson HD, Tyne K, Bougatsos C, Chan BK, Humphrey L (2009). Screening for breast cancer: an update for the U.S. Preventive Services Task Force. Ann Intern Med.

[9] Frosch DL, Elwyn G (2010). I believe, therefore I do (Editorial). J Gen Intern Med.

[10] Kiviniemi MT, Hay JL (2012). Awareness of the 2009 US preventive services task force guidelines in women ages 40–49 and 50. BMC Public Health.

[11] Stacey D, Légaré F, Col NF, Bennett CL, Barry MJ, Eden KB (2014). Decision aids for people facing health treatment or screening decisions. Cochrane Database Syst Rev.

[12] Elwyn G, O'Connor AM, Bennett C, Newcombe RG, Politi M, Durand MA (2009). Assessing the quality of decision support technologies using the International Patient Decision Aid Standards instrument. PLoS ONE.

[13] Krebs P, Prochaska JO, Rossi JS (2010). A meta-analysis of computer-tailored interventions for health behavior change. Prev Med.

[14] Joseph-Williams N, Evans R, Edwards A, Newcombe RG, Wright P, Grol R (2010). Supporting informed decision making online in 20 min: an observational web-log study of a PSA test decision aid. J Med Internet Res.

[15] American Cancer Society (2013). Breast cancer facts & figures.

[16] International Patient Decision Aids Standards (IPDAS) Collaboration [Document on the internet], http://ipdas.ohri.ca/IPDAS_checklist.pdf. Accessed January 21, 2015.

[17] Qaseem A, Snow V, Sherif K, Aronson M, Weiss KB, Owens DK (2007). Screening mammography for women 40 to 49 years of age: a clinical practice guideline from the American College of Physicians. Ann Intern Med.

[18] Armstrong K, Moye E, Williams S, Berlin JA, Reynolds EE (2007). Screening mammography in women 40 to 49 years of age: a systematic review for the American College of Physicians. Ann Intern Med.

[19] Amir E, Freedman OC, Seruga B, Evans D (2010). Assessing women at high risk of breast cancer: a review of risk assessment models. J Natl Cancer Inst.

[20] Scariati P (2011). Making choices about breast cancer screening: a decision aid for women between the ages of 38 and 48.

[21] O'Connor AM (1995). Validation of a decisional conflict scale. Med Decis Making.

[22] Linder SK, Swank PR, Vernon SW, Mullen PD, Morgan RO, Volk RJ (2011). Validity of a low literacy version of the decisional conflict scale. Patient Educ Couns.

[23] Kryworuchko J, Stacey D, Bennett C, Graham ID (2008). Appraisal of primary outcome measures used in trials of patient decision support. Patient Educ Couns.

[24] O’Connor A. User manual—Decisional Conflict Scale (ten item question format) [document on the internet]. Ottawa: Ottawa Hospital Research Institute, 1993. updated 2010. http://decisionaid.ohri.ca/docs/develop/User_Manuals/UM_Decisional_Conflict.pdf. Accessed January 21, 2015.

[25] O’Connor A. User manual – Decision self-efficacy scale [document on the internet]. Ottawa: Ottawa hospital Research Institute, 1995a. http://decisionaid.ohri.ca/docs/develop/User_Manuals/UM_Decision_SelfEfficacy.pdf . Accessed January 21, 2015.

[26] Stacey C, O’Connor A, DeGrasse C, Verna S (2003). Development and evaluation of a breast cancer prevention decision aid for higher risk women. Health Expect.

