# Supplementary material for: Impact of a decision aid on reducing uncertainty: pilot study of women in their 40s and screening mammography
Source: BMC Med Inform Decis Mak. 2015 Nov 10;15:89. doi: 10.1186/s12911-015-0210-2 (PMC4640415; doi:10.1186/s12911-015-0210-2)
Supplement: Additional file 1: — Screen shots depicting the Values Clarification section of the Mammography Screening Decision Aid. (PDF 332 kb) [file 12911_2015_210_MOESM1_ESM.pdf]

Additional file 1; Screen shots depicting the Values Clarification section of the Mammography Screening Decision Aid

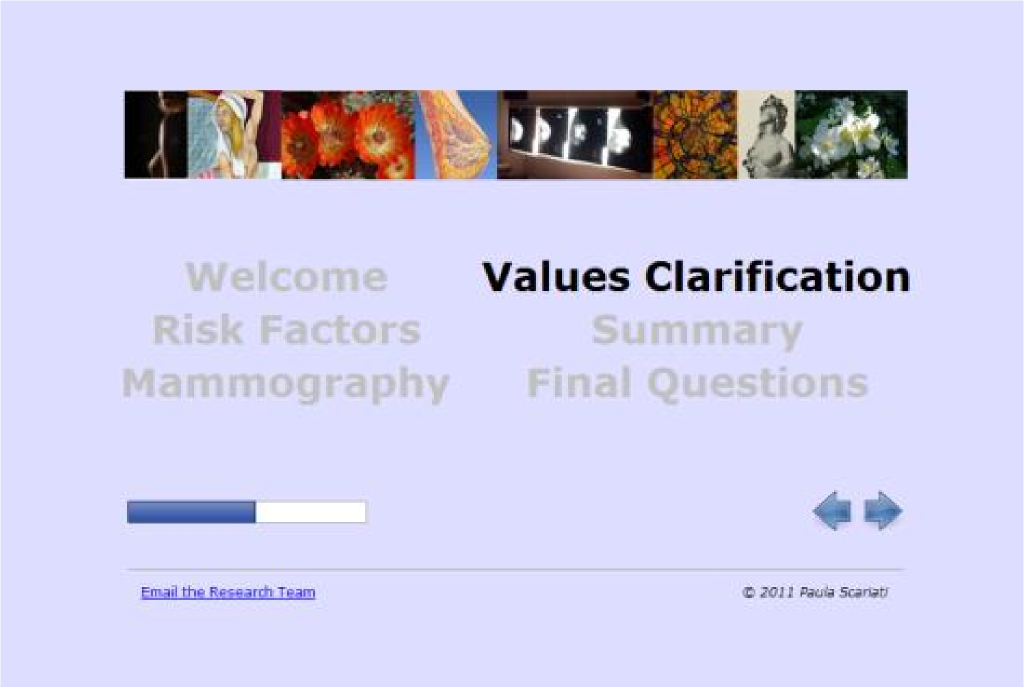

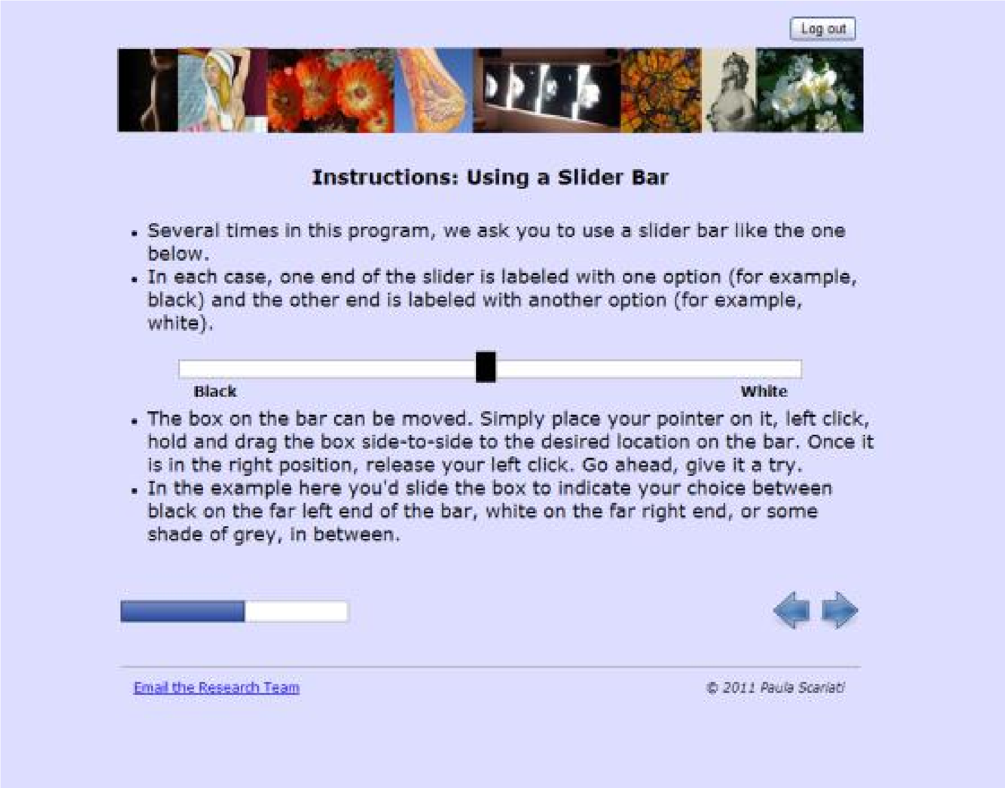

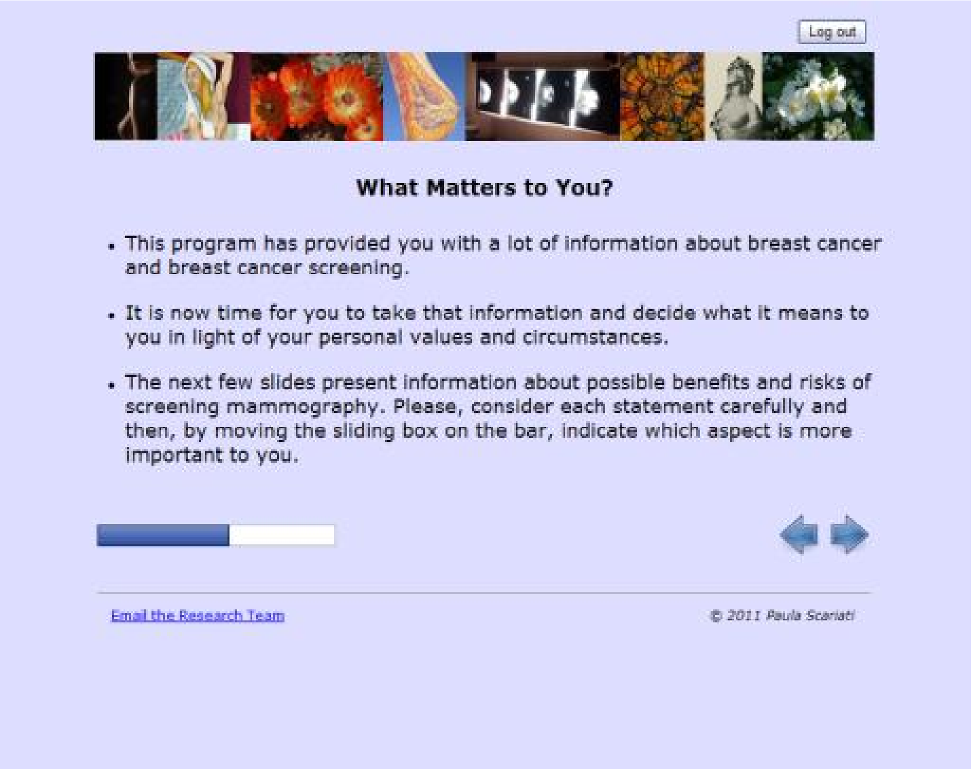
Log out
What Matters to You?
This program has provided you with a lot of information about breast cancer and breast cancer screening.
It is now time for you to take that information and decide what it means to you in light of your personal values and circumstances.
The next few slides present information about possible benefits and risks of screening mammography. Please, consider each statement carefully and then, by moving the sliding box on the bar, indicate which aspect is more important to you.
Email the Research Team
© 2011 Paula Scariati

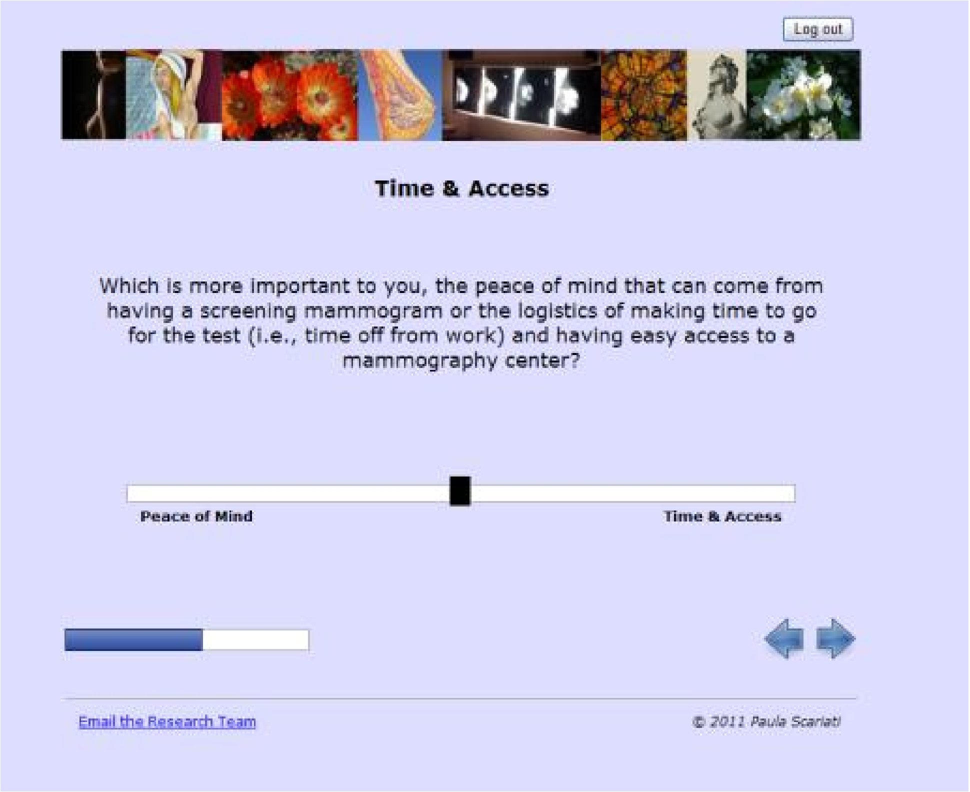
Log out
Time & Access
Which is more important to you, the peace of mind that can come from having a screening mammogram or the logistics of making time to go for the test (i.e., time off from work) and having easy access to a mammography center?
Peace of Mind
Time & Access
Email the Research Team
© 2011 Paula Scariati

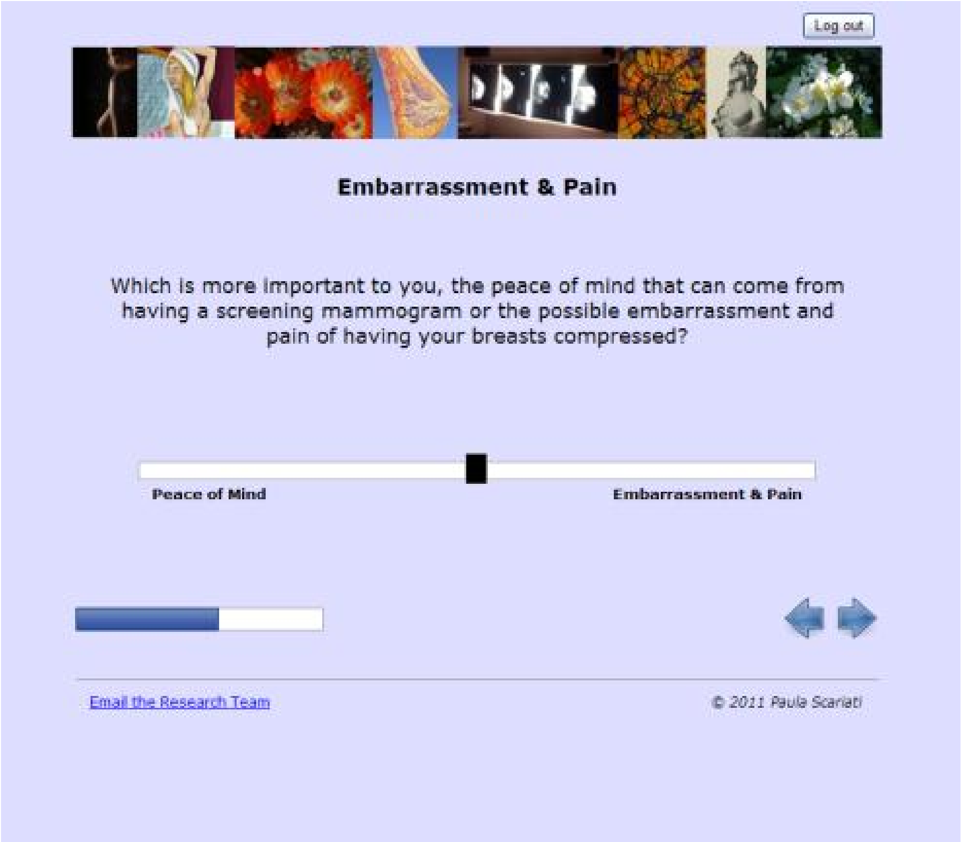
Log out
Embarrassment & Pain
Which is more important to you, the peace of mind that can come from having a screening mammogram or the possible embarrassment and pain of having your breasts compressed?
Peace of Mind
Embarrassment & Pain
Email the Research Team
© 2011 Paula Scariati

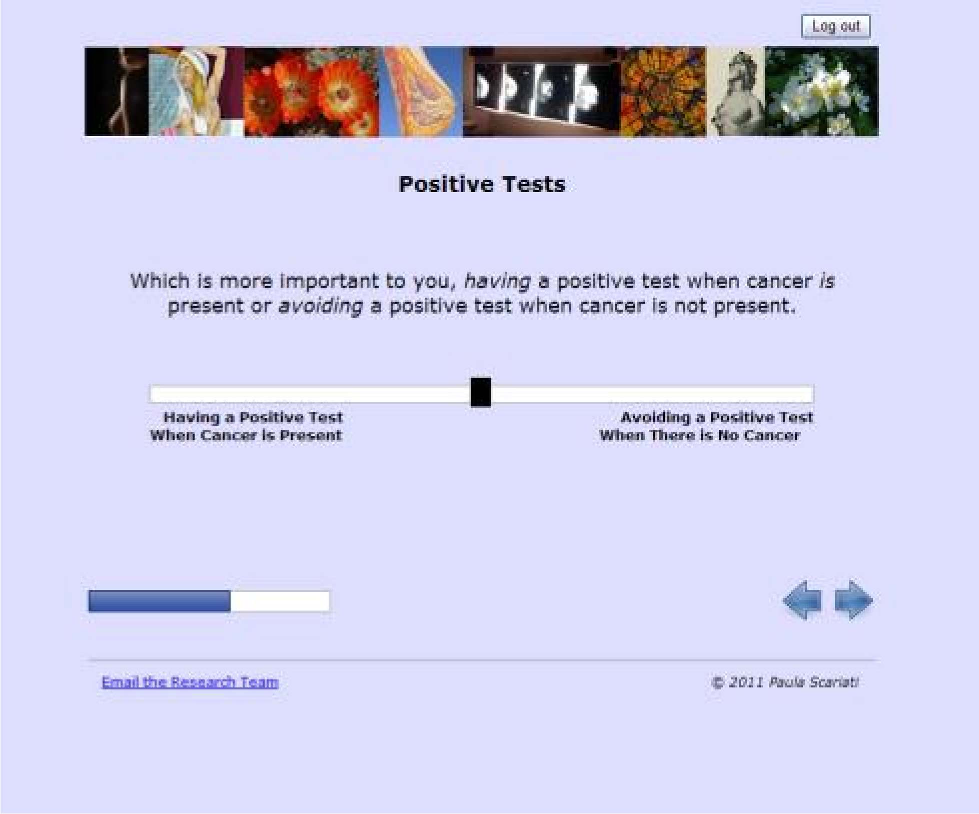
Log out
Positive Tests
Which is more important to you, *having* a positive test when cancer *is* present or *avoiding* a positive test when cancer is not present.
Having a Positive Test When Cancer is Present
Avoiding a Positive Test When There is No Cancer
Email the Research Team
© 2011 Paula Scariati

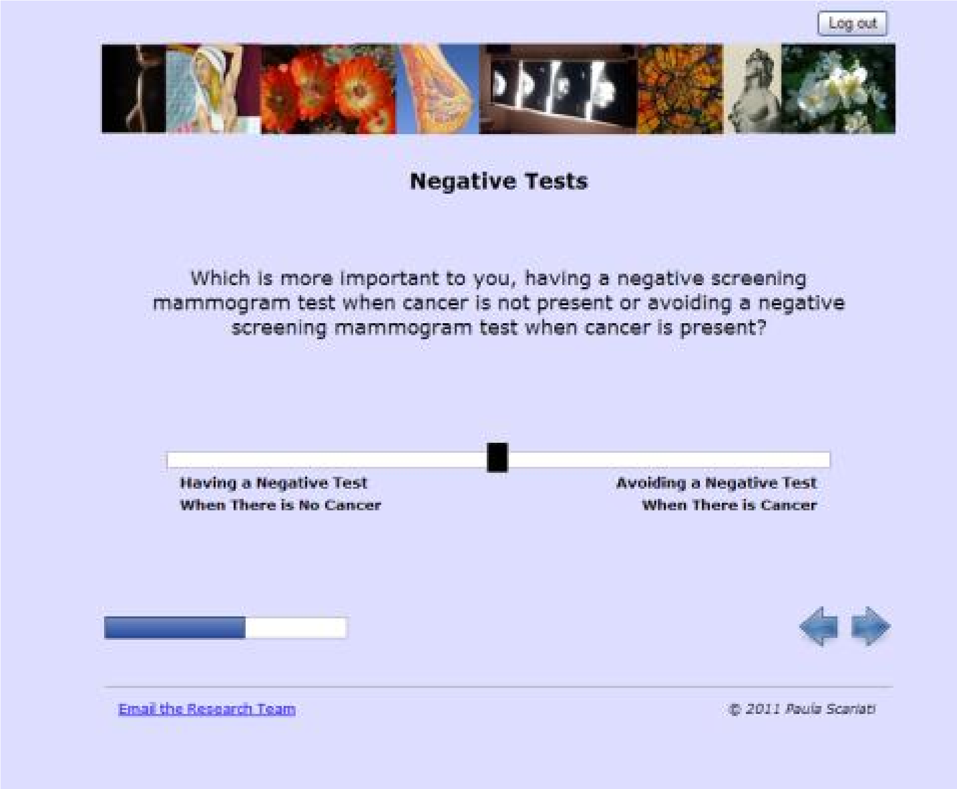
Log out
Negative Tests
Which is more important to you, having a negative screening mammogram test when cancer is not present or avoiding a negative screening mammogram test when cancer is present?
Having a Negative Test When There is No Cancer
Avoiding a Negative Test When There is Cancer
Email the Research Team
© 2011 Paula Scariati

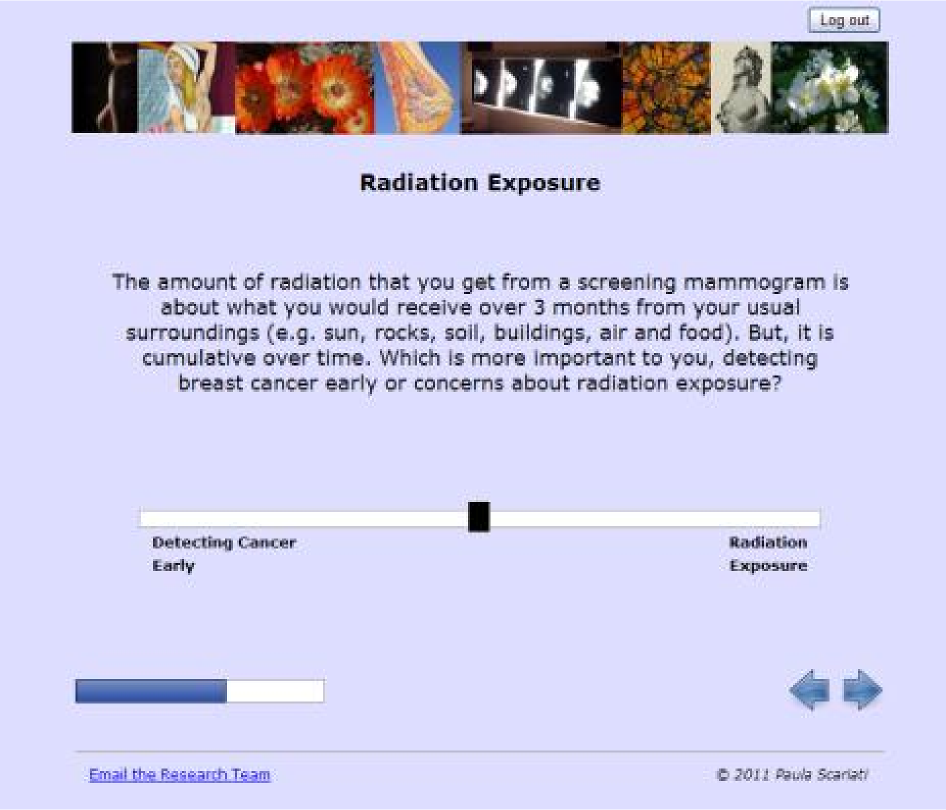
Log out
Radiation Exposure
The amount of radiation that you get from a screening mammogram is about what you would receive over 3 months from your usual surroundings (e.g. sun, rocks, soil, buildings, air and food). But, it is cumulative over time. Which is more important to you, detecting breast cancer early or concerns about radiation exposure?
Detecting Cancer Early
Radiation Exposure
Email the Research Team
© 2011 Paula Scariati

Log out

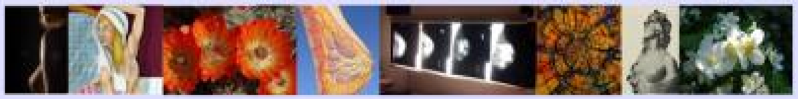

Overdiagnosis

Screening mammograms may detect cancers that would never go on to create further problems. This is called overdiagnosis. Which is more important to you, detecting a cancer that could be fatal or avoiding the detection of a cancer that would never be fatal?

Detect a Cancer that Could be Fatal | **Avoid Detecting a Cancer that Would Not be Fatal**

Email the Research Team

© 2011 Paula Scariati

Log out

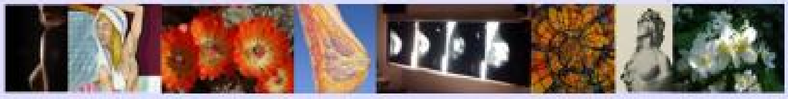

Stress & Fear

Which is more important to you, the peace of mind that can come from having a screening mammogram or the potential stress & fear that can result if follow-up testing is required?

Peace of Mind | **Stress & Fear**

Email the Research Team

© 2011 Paula Scariati

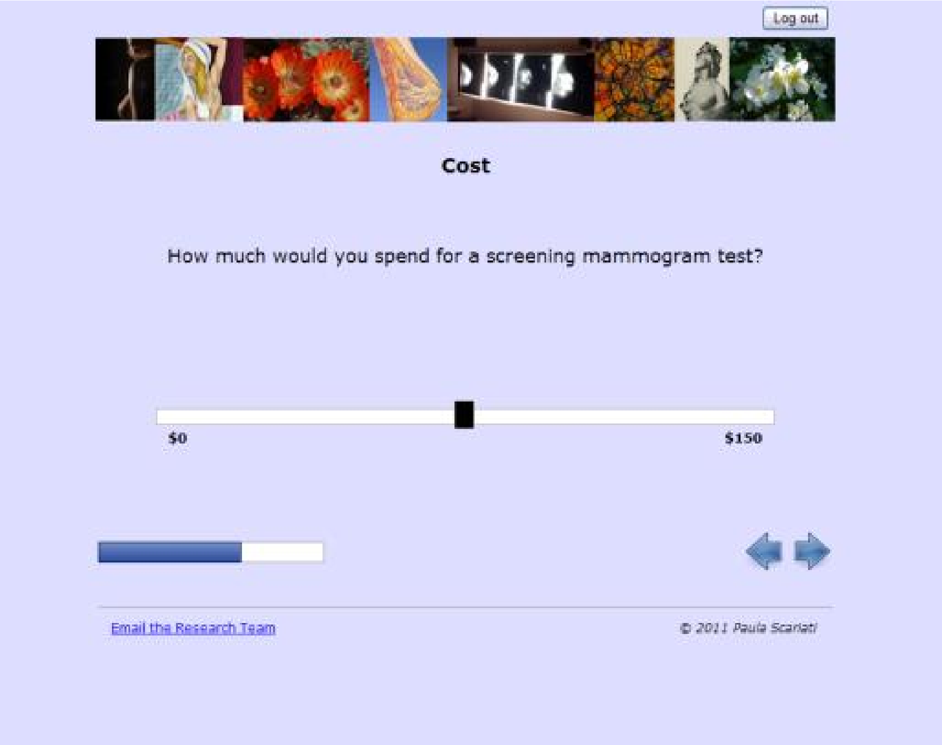
Log out
Cost
How much would you spend for a screening mammogram test?
$0
$150
Email the Research Team
© 2011 Paula Scariati

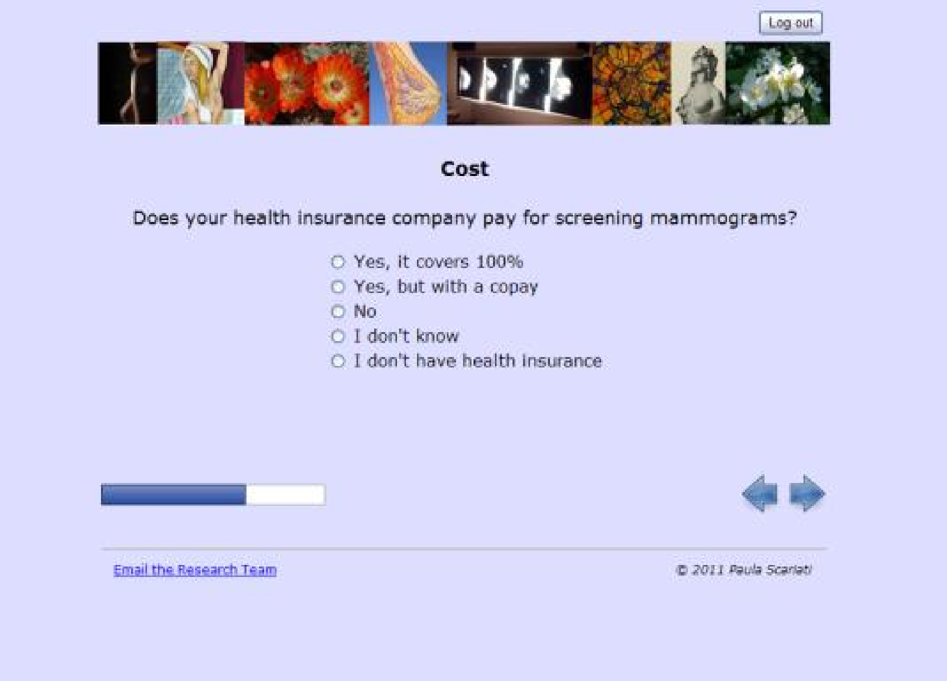
Log out
Cost
Does your health insurance company pay for screening mammograms?
Yes, it covers 100%
Yes, but with a copay
No
I don't know
I don't have health insurance
Email the Research Team
© 2011 Paula Scariati

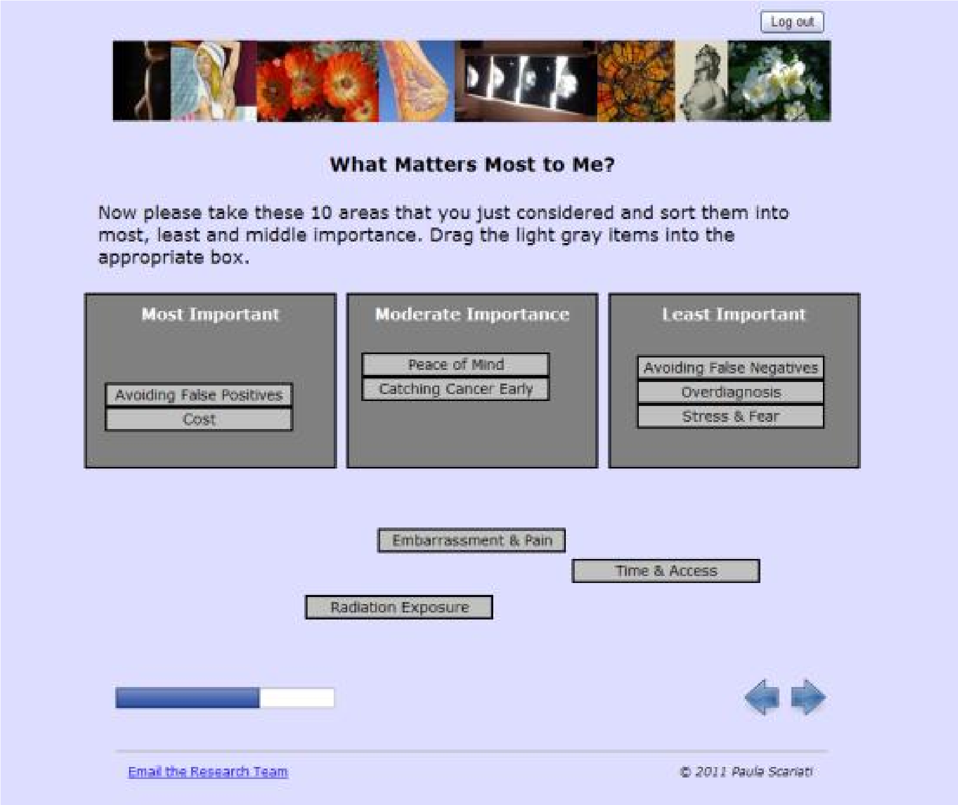
Log out
What Matters Most to Me?
Now please take these 10 areas that you just considered and sort them into most, least and middle importance. Drag the light gray items into the appropriate box.
Most Important
Avoiding False Positives
Cost
Moderate Importance
Peace of Mind
Catching Cancer Early
Least Important
Avoiding False Negatives
Overdiagnosis
Stress & Fear
Embarrassment & Pain
Time & Access
Radiation Exposure
Email the Research Team
© 2011 Paula Scariati
